# Clinical Characteristics and Prevalence of Comorbidities according to Metformin Use in Korean Patients with Type 2 Diabetes

**DOI:** 10.1155/2020/9879517

**Published:** 2020-07-24

**Authors:** Sang Ouk Chin, In Gyoon Ha, Sang Youl Rhee, Su Jin Jeong, Suk Chon, Sung Hoon Kim, Kyu Jeung Ahn, Sei Hyun Baik, Yongsoo Park, Moon Suk Nam, Kwan Woo Lee, Jeong Taek Woo

**Affiliations:** ^1^Department of Endocrinology and Metabolism, College of Medicine, Kyung Hee University, Seoul, Republic of Korea; ^2^Statistics Support Department, Kyung Hee University, Medical Center Science Research Institute, Seoul, Republic of Korea; ^3^Division of Endocrinology and Metabolism, Department of Medicine, Cheil General Hospital and Women's Healthcare Center, College of Medicine, Dankook University, Yongin, Republic of Korea; ^4^Division of Endocrinology and Metabolism, Department of Internal Medicine, College of Medicine, Korea University, Seoul, Republic of Korea; ^5^Department of Internal Medicine, College of Medicine, Hanyang University, Seoul, Republic of Korea; ^6^Department of Internal Medicine, College of Medicine, Inha University, Incheon, Republic of Korea; ^7^Department of Endocrinology and Metabolism, College of Medicine, Ajou University, Suwon, Republic of Korea

## Abstract

**Methods:**

This cross-sectional study based on the Korean National Diabetes Program 2 registry used its baseline clinical data collected from seven participating university hospitals in Korea. Patients with no significant changes in their oral hypoglycemic agents and no diabetes-related complications within the year prior to participation were enrolled. Patients' clinical characteristics according to metformin use were analyzed.

**Results:**

Among 858 subjects included in the analyses, 706 were metformin users and 152 were nonmetformin users. Metformin users were significantly younger and had higher and glycated hemoglobin with significantly lower rates of accompanying microvascular complications such as retinopathy, cataracts, overt proteinuria, renal insufficiency, and peripheral neuropathy than nonusers. Meanwhile, there was a significantly lower prevalence of malignancy and depression among metformin users. These associations remained significant in multivariate analyses. The prevalence rate of macrovascular complications was not significantly different between the two groups.

**Conclusions:**

There were significant differences with respect to clinical characteristics and comorbidity prevalence according to metformin use among Korean type 2 diabetes patients. Long-term follow-up of these patients is necessary to observe how this difference will affect clinical outcomes for these patients.

## 1. Introduction

The prevalence of type 2 diabetes in Korea is reported to be 12.6% in adults over age 30, according to the 2011 Korea National Health and Nutritional Examination Survey, which was based on a nationwide cross-sectional health surveillance program [1]. With its increasing prevalence, the medical care cost and the acute and chronic complication rates for diabetes should also likely increase.

Many clinical guidelines and consensus statements currently recommend metformin in combination with lifestyle management as the first treatment option for patients with newly diagnosed type 2 diabetes patients, if not contraindicated [2, 3]. Metformin has been reported to offer the additional benefit of preventing cardiovascular diseases (CVD) [4], although this result was disputed in a recent meta-analysis [5]. Since metformin is eliminated by the kidneys [6], traditionally it has not been recommended for patients with moderate-to-severe renal insufficiency due to the possibility of lactic acidosis [7]. However, a recent study and meta-analysis demonstrated that metformin can be safely used in patients with mild-to-moderate chronic kidney disease, showing a similar incidence rate for lactic acidosis regardless of renal function [8, 9], which expands the potential range of patients that can be helped by metformin [2]. In addition to renal insufficiency, guidelines indicate other conditions in which metformin should not be used when treating patients with diabetes.

This means, despite the core role of metformin as a first-line treatment option as recommended in many guidelines, it is very common to avoid metformin and to prescribe other oral hypoglycemic agents when treating patients. Therefore, an investigation of patients' characteristics and their accompanying morbidities according to metformin use would help us to identify the unique features of two groups, which may enable us to improve our ability to provide individualized and effective treatment plans as well as to predict prognosis. Thus, this study was aimed to compare patient characteristics according to metformin use among the KNDP2 cohort study, which is a representative cohort of patients with diabetes in Korea.

## 2. Methods

### 2.1. KNDP2 Cohort Study

The Korean National Diabetes Program 2 (KNDP2) cohort study was a follow-up study of the KNDP study. The KNDP study was conducted from 2006 to 2011 and used a prospective, multicenter, observational design. Details of the study were described previously [10]. Using an identical design to evaluate Korean patients with type 2 diabetes, the KNDP2 cohort study aimed to establish clinical research infrastructure encompassing local clinics and university hospitals that enabled investigators to collect and analyze clinical data from patients with type 2 diabetes. Participating hospitals included Kyung Hee University Hospital at Gangdong, Cheil General Hospital and Women's Healthcare Center, Korea University Guro Hospital, Hanyang University Guri Hospital, Inha University Hospital, and Ajou University Hospital.

### 2.2. Subjects and Study Design

We conducted a cross-sectional study using KNDP2 registry data collected from the seven participating university hospitals. From May 2014 to March 2015, 858 patients with type 2 diabetes between ages 20 and 80 years with no diabetes-related acute or chronic complications within three months prior to KNDP2 enrollment were recruited into our study. Clinical data, including blood and urine samples, were collected via an electronic case report form (eCRF) from each participant after obtaining informed consent. The eCRF included critical clinical items, as recommended in the guidelines published by the American Diabetes Association and the Korean Diabetes Association (KDA) [11, 12], and was developed after review and comments from investigators who participated in the KNDP2 study. The eCRF items were also endorsed by an expert group from the KDA, as described previously [13]. Based on this eCRF, common data elements (CDEs) and standard operating procedures (SOPs) were developed to secure standardized clinical and biochemical data and to establish practical action plans.

The eCRF includes the following information collected from interviews conducted during cohort recruitment and from medical record reviews: age, anthropometrics, and duration of diabetes comorbidities including hypertension, dyslipidemia, cancer, autoimmune diseases, tuberculosis, depression, periodontal disease, and macro-/microvascular complications. Medications included in the analysis were defined when satisfying the following criteria: oral hypoglycemic agents/insulin, antihypertensive drugs, or statins with a prescription history of >2 months in length and within 3 months of recruitment. Laboratory data were also collected at recruitment and included in the eCRF: fasting blood glucose, glycated hemoglobin A1c (HbA1c), total cholesterol (T-chol), triglycerides (TG), low-density lipoprotein cholesterol (LDL-C), high-density lipoprotein cholesterol (HDL-C), blood urea nitrogen (BUN), serum creatinine (Cr), and urine microalbumin. Fasting blood glucose levels were measured using the hexokinase method after blood samples were centrifuged. HbA1c levels were determined using the high-performance liquid chromatography method that received National Glycohemoglobin Standardization Program certification. T-chol, TG, HDL-C, LDL-C, BUN, and Cr were analyzed using an automated analyzer that received a certification of quality control from the Korean Society for Laboratory Medicine. These clinical characteristics of enrolled patients from the eCRF were cross-sectionally collected and compared according to metformin use at the time of data collection.

### 2.3. Statistics

**Figure 1:**
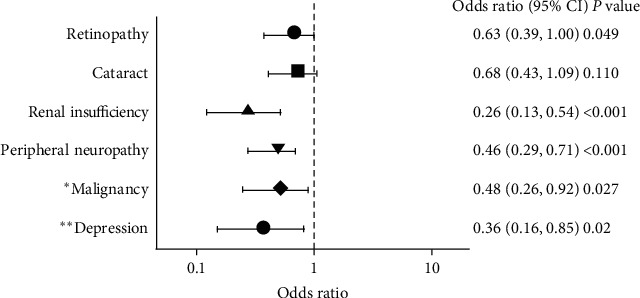
Multivariate logistic regression analysis to observe the association between metformin use and comorbidities adjusted by age, sex, BMI, HbA1c total cholesterol, history of hypertension/hyperlipidemia, medication for diabetes, antiplatelet medication, family history of comorbidity, smoking status, alcohol use, creatinine, and creatinine clearance. Malignancy adjusted by age, sex, family history of malignancy, smoking status, HbA1c, and diabetes duration. Depression adjusted by age, sex, history of stroke, family history of depression, and diabetes duration.

Patients were stratified by metformin use, and their clinical characteristics were analyzed. Descriptive values are expressed as means and standard deviations, and Student's *t*-test and the chi-square test were used to evaluate statistical significance. *P* values < 0.05 were considered significant. Logistic regression analysis was used to estimate correlations between the two groups. Univariate logistic regression was performed to estimate correlations for each variable and disease, and multiple logistic regression was performed to adjust for confounding variables, such as age, sex, and others (Figure 1 for the detailed explanation). Statistical analyzes were performed by a statistician at the Kyung Hee University Medical Science Research Institute. SAS software version 9.3 (SAS Institute Inc., Cary, NC, USA) was used to perform all statistical tests.

### 2.4. Ethics Statement

This study was approved by the Institutional Review Board of Kyung Hee University Hospital (KMC IRB 1428-03). Written informed consent was obtained from all participants. Data from this study are registered at the Clinical Research Information Service (CRIS, No. KCT0001232). The CRIS is a Korean national service connected to the International Clinical Trials Registry Platform of the World Health Organization.

## 3. Results

### 3.1. Baseline Characteristics

Table 1 shows baseline clinical characteristics of subjects according to metformin use. A total of 857 patients were enrolled in the study, of whom 706 (82.3%) were taking metformin and 151 (17.7%) were not. Other hypoglycemic agents used in either the metformin user group or nonuser group are listed in Table 2. There was no significant difference in sex proportions between the two groups. The mean age of the metformin user group was significantly younger, and they had shorter durations of diabetes than the nonuser group. The metformin users had significantly higher BMI, body weight, waist circumference, and hip circumference than metformin nonusers. Serum creatinine was significantly lower, and creatinine clearance was significantly higher in the metformin user group. The metformin user group also had significantly higher HbA1c and lower serum creatinine and LDL cholesterol levels.

### 3.2. Comparison of Comorbidities

There was a significant difference in comorbidities between the metformin user and nonuser groups (Table 3). The prevalence of cancer (*n* = 38 (5.43%) in the metformin user group, and *n* = 17 (11.3%) in the nonuser group) and depression (*n* = 18 (2.57%) in the metformin user group, and *n* = 9 (6.0%) in the nonuser group) was significantly lower in the metformin user group, according to chi-square analysis (*P* = 0.016 for cancer, and *P* = 0.039 for depression), and these results remained significant after adjusting for age and sex.

### 3.3. Comparison of Macro- and Microvascular Complications of Diabetes

Tables 4 and 5 show the prevalence of macro- and microvascular complications of diabetes according to metformin use. While no significant difference was observed in macrovascular complications according to metformin use (Table 4), the prevalence of diabetic retinopathy, cataracts, overt proteinuria, renal insufficiency (estimated glomerular filtration rate < 60 mL/min/1.73 m^2^), and peripheral neuropathy was lower in the metformin user group (Table 5). To evaluate the independent association between metformin use and comorbidities, multivariate logistic regression analysis was performed with correction for sex, age, and other selected variables (Figure 1). Odds ratios for various comorbidities among metformin users compared with nonusers were as follows: retinopathy, OR = 0.63 (95% CI: 0.39–1.00); renal insufficiency, OR = 0.26 (95% CI: 0.13–0.54); peripheral neuropathy, OR = 0.46 (95% CI: 0.29–0.71); malignancy, OR = 0.48 (95% CI: 0.26–0.92); and 0.36 (95% CI: 0.16–0.85).

## 4. Discussion

This cross-sectional study compared the clinical characteristics of patients with type 2 diabetes according to metformin use. Results indicate that the metformin user group had a significantly lower prevalence of accompanying microvascular complications and other comorbidities, such as cancer, compared with nonusers. However, there was no significant difference in prevalence of macrovascular complications between the metformin user and nonuser groups. These findings suggest that metformin nonusers have a higher risk of adverse outcomes for their prognosis, thus requiring more careful management of their diabetes.

Among our study patients, the metformin nonusers tended to be older but also had lower BMI and waist circumference, which may have been possibly due to relatively poor general condition associated with a longer duration of diabetes in these patients (12.61 ± 7.23 vs 14.16 ± 8.12 years*, P* = 0.020, Table 1). Renal function was also significantly deteriorated among the nonuser group, and they had higher prevalence of microvascular complications such as proteinuria and renal insufficiency, which was reflected in their recent prescription patterns based on guidelines limiting metformin use in patients with impaired renal function [2, 14]. The prevalence of diabetic retinopathy and neuropathy was also higher in the metformin nonuser group, which is consistent with previous findings of an association between diabetes duration and microvascular complications [15, 16].

Our study demonstrated that prevalence of cancer, depression, and periodontal disease was significantly higher in the metformin nonuser group. In addition, hypertension, hyperlipidemia, and tuberculosis were more prevalent in the nonuser group but not significantly. Patients with diabetes are known to be more likely to have a higher risk of accompanying a depressive condition, and people with depression are shown to have an increased risk of developing diabetes [17]. This bidirectional association between diabetes and depression would aggravate in those with poor glucose control [18]. This relationship is shown to be additive, which leads to a higher mortality than patients with diabetes or depression alone [19]. Patients with both diseases also are reported to have a higher risk to experience microvascular complications and periodontal disease [20, 21]. As for cancer and diabetes, many epidemiologic studies have reported an elevated cancer risk among patients with diabetes [22–27]. In contrast, metformin is thought to have anticancer effects, according to previous in vitro studies [28–33]. Also, it has been reported that patients with diabetes accompanying depression or periodontal disease may expect further improvement by using metformin [34, 35]; patients who do not use metformin to treat their diabetes will not experience these additional benefits that metformin users may be expected to experience, though it is not clear why some comorbidities were significantly more common among nonusers. Therefore, it is important to pay close attention to metformin nonusers by applying a more individualized pharmacological approach and emphasizing the importance of lifestyle modification for better glucose control and to prevent deterioration of accompanied diseases.

Previous studies which investigated the effects of metformin on cardiovascular outcomes have produced variable results. UKPDS34, which evaluated the effects of metformin on overweight patients with type 2 diabetes, found a reduction in the risk of nonfatal myocardial infarction [4]. However, two meta-analyses reported an increase in cardiovascular risk when metformin was used with sulfonylurea [36, 37], furthering the debate regarding metformin's effect on cardiovascular outcomes. On the contrary, a number of previous studies reported that sulfonylurea may increase the cardiovascular risk, especially when compared with metformin [38, 39]. The metformin users were shown to use sulfonylurea more frequently than nonusers (Table 2), which may have offset the cardiovascular benefit of metformin leading to the lack of difference in macrovascular complications between two groups (Table 4). Considering the practical difficulty in conducting a large-scale clinical study of metformin use, future long-term observation of our study population could yield more valuable data about the cardiovascular effects of metformin.

A major limitation of this study is its cross-sectional design. Therefore, caution is necessary to avoid misinterpreting our results as having a causal relationship. Especially for depression, it is hard to draw the conclusion that use of metformin led to a lower prevalence of depression when considering our study design. Previous studies demonstrated the negative effect of depression, which can aggravate patient's glucose control [34] and the promising role of metformin on improvement of cognitive functions in an animal study by inducing anti-inflammatory effects [40] and promoting neurogenesis in hippocampus [41]. This study, by reviewing the real clinical data, indirectly confirmed the previously published results, in which metformin can demonstrate beneficial effects on patients with diabetes and depression. In addition, this study was based on carefully controlled multicenter cohort data that utilized CDEs and SOPs for robust analyses. It should be noted that few studies have investigated clinical characteristics of cohort participants according to metformin use. Also, the authors were not able to collect the data regarding the duration of metformin use, possible history of using metformin in the metformin nonuser group, and the reason for not using metformin in the nonuser group, as well as the duration after which the complications occurred. Last, sodium-glucose transport protein 2 (SGLT2) inhibitors and GLP-1 (glucagon-like peptide-1) agonists well-known for their benefits on the cardiovascular outcome according to large-scaled trials [42, 43] were shown to be rarely used in our KNDP2 cohort. It is because these two drugs had been newly introduced in Korea at the time of data collection and thus were not being commonly prescribed when compared with present time.

In conclusion, significant differences in clinical characteristics according to metformin use were observed among patients with diabetes. In particular, metformin nonusers had higher prevalence of accompanying comorbidities including cancers and microvascular complications such as retinopathy, peripheral neuropathy, and cataracts. We are currently investigating clinical outcomes among our cohort participants according to metformin use to observe the effect of metformin on future prognoses.

## Figures and Tables

**Table 1:** Difference of baseline characteristics of subjects according to the use of metformin.

Variable	Metformin user	Metformin nonuser	*P*
*n*	706 (82.3%)	152 (17.7%)	
Sex, male/female			0.210
Male, *n* (%)	321 (80.5%)	78 (19.5%)	
Female, *n* (%)	385 (83.9%)	74 (16.1%)	
Age, years old	59.42 ± 9.77	63.16 ± 9.87	<0.001
Duration of diabetes, years	12.61 ± 7.23	14.16 ± 8.12	0.020
Body weight, kg	66.75 ± 11.68	64.39 ± 11.16	0.023
Body mass index, kg/m^2^	25.44 ± 3.46	24.67 ± 3.23	0.013
Waist circumference, cm	89.02 ± 8.73	86.56 ± 7.93	0.004
Chest circumference, cm	94.49 ± 7.27	92.69 ± 7.43	0.042
Hip circumference, cm	95.2 ± 6.55	93.21 ± 6.44	0.002
Glycosylated hemoglobin, %	7.45 ± 1.27	7.1 ± 1.37	0.002
Fasting plasma glucose, mg/dL	150 ± 49.62	140.88 ± 56.09	0.063
Urine microalbumin, *μ*g/mg Cr	39.04 ± 110.86	50.46 ± 141.85	0.371
Serum creatinine, mg/dL	0.82 ± 0.28	1.11 ± 1.35	0.010
Creatinine clearance, ml/min	96.52 ± 28.8	84.5 ± 30.98	<0.001
ALT, IU/L	23.24 ± 23.00	24.29 ± 15.63	0.011
Total cholesterol, mg/dL	161.71 ± 32.86	173.29 ± 36.64	<0.001
Triglyceride, mg/dL	146.92 ± 87.65	133.75 ± 73.27	0.056
LDL cholesterol, mg/dL	89.73 ± 28.84	100.5 ± 31.22	<0.001
HDL cholesterol, mg/dL	48.44 ± 13.07	51.51 ± 15.65	0.027

ALT, alanine transaminase; LDL, low-density lipoprotein; and HDL, high-density lipoprotein. Results are expressed as mean ± standard deviation or *n* (%). *P* value for the *t*-test comparing the both groups with and without metformin.

**Table 2:** Difference in the use of medications according to the use of metformin.

Variable	Metformin user	Metformin nonuser	*P*
Sulfonylurea	312 (44.2)	48 (31.6)	0.004
DPP-4 inhibitor	279 (39.5)	36 (23.7)	0.002
Meglitinide	12 (1.7)	9 (5.9)	0.006
Thiozolidinedione	32 (4.5)	9 (5.9)	0.698
*α*-Glucosidase inhibitor	11 (1.6)	9 (5.9)	0.004
SGLT2 inhibitor	0	0	
GLP-1 agonist	2 (0.3)	0	1.000
Rapid-acting insulin	20 (2.8)	11 (7.2)	0.008
Long-acting insulin	92 (13.0)	27 (17.8)	0.125
Premixed insulin	60 (8.5)	20 (13.16)	0.073

Values are expressed as number (%). *P* value for the chi-square test comparing the groups with and without metformin. DPP-4, dipeptidyl peptidase-4; SGLT2, sodium-glucose transporter protein 2; and GLP-1, glucagon-like peptide-1.

**Table 3:** Difference of comorbidity according to the use of metformin.

Variable	Metformin user	Metformin nonuser	*P*
Hypertension, *n* (%)	417 (59.32%)	101 (66.45%)	0.120
Hypertension duration, years	5.67 ± 7.24	8.17 ± 9.97	0.004
Dyslipidemia, *n* (%)	436 (62.02%)	97 (63.82%)	0.713
Dyslipidemia duration, years	4.44 ± 5.22	5.50 ± 6.21	0.053
Cancer, *n* (%)	38 (5.43%)	17 (11.33%)	0.016
Cancer duration, years	0.40 ± 1.96	1.15 ± 3.58	0.023
Autoimmune disease, *n* (%)	17 (2.43%)	2 (1.33%)	0.553
Tuberculosis, *n* (%)	21 (3%)	9 (6%)	0.086
Depression, *n* (%)	18 (2.57%)	9 (6%)	0.039
Periodontal disease, *n* (%)	72 (10.29%)	26 (17.33%)	0.023

Results are expressed as mean ± standard deviation or *n* (%). *P* value for the *t*-test or the chi-square test comparing both the groups with and without metformin.

**Table 4:** Difference of macrovascular complication of diabetes according to the use of metformin.

Variable	Metformin user	Metformin nonuser	*P*
Myocardial infarction, *n* (%)	9 (1.28%)	2 (1.32%)	1.000
Angina, *n* (%)	55 (7.58%)	15 (9.93%)	0.414
PTCA, *n* (%)	7 (1%)	1 (0.66%)	1.000
PCI with stent insertion, *n* (%)	27 (3.85%)	8 (5.3%)	0.374
CABG, *n* (%)	4 (0.57%)	0 (0%)	1.000
Peripheral artery disease, *n* (%)	4 (0.57%)	0 (0%)	1.000

PTCA, percutaneous transluminal coronary angioplasty; PCI, percutaneous coronary intervention; and CABG, coronary artery bypass graft surgery. Results are expressed as mean ± standard deviation or *n* (%). *P* value for the *t*-test or the chi-square test comparing the both groups with and without metformin.

**Table 5:** Difference of microvascular complication of diabetes according to the use of metformin.

Variable	Metformin user	Metformin nonuser	*P*
Diabetic retinopathy, *n* (%)	136 (19.4%)	44 (29.14%)	0.011
DR duration, years	1.31 ± 3.31	2.33 ± 4.30	0.007
Photocoagulation, *n* (%)	28 (4.01%)	14 (9.27%)	0.012
Duration, years	0.29 ± 1.68	0.60 ± 2.43	0.131
Intravitreal injection, *n* (%)	20 (2.86%)	12 (7.95%)	0.007
Duration, years	0.17 ± 1.07	0.55 ± 2.09	0.028
Ophthalmic surgery, *n* (%)	82 (11.73%)	30 (19.87%)	0.011
Duration, years	0.78 ± 2.79	1.50 ± 3.62	0.023
Cataract, *n* (%)	135 (19.26%)	50 (33.11%)	<0.001
Duration, years	1.05 ± 2.99	2.31 ± 4.64	0.002
Overt proteinuria, *n* (%)	31 (4.4%)	16 (10.53%)	0.005
Duration, years	0.25 ± 1.33	0.75 ± 2.50	0.018
Renal insufficiency, *n* (%)	37 (5.25%)	29 (19.08%)	<0.001
Duration, years	0.36 ± 1.68	1.22 ± 3.01	<0.001
Peripheral polyneuropathy, *n* (%)	169 (24.0%)	63 (41.45%)	<0.001
Duration, years	1.48 ± 3.07	3.13 ± 4.55	<0.001

DR, diabetic retinopathy; PPN, peripheral polyneuropathy; Results are expressed as mean ± standard deviation or *n* (%). *P* value for *t*-test or chi-square test comparing both the groups with and without metformin.

## Data Availability

The data utilized and analyzed to support the findings of our study are included within the article.
